# C-Command in the Grammars of Children with High Functioning Autism

**DOI:** 10.3389/fpsyg.2017.00402

**Published:** 2017-03-28

**Authors:** Neha Khetrapal, Rosalind Thornton

**Affiliations:** ^1^Department of Linguistics, Language Acquisition Research Group, Macquarie UniversitySydney, NSW, Australia; ^2^ARC Centre of Excellence in Cognition and its Disorders, Macquarie UniversitySydney, NSW, Australia

**Keywords:** grammatical development, reflexives, negation, disjunction, c-command

## Abstract

A recent study questioned the adherence of children with Autism Spectrum Disorders (ASD) to a linguistic constraint on the use of reflexive pronouns (Principle A) in sentences *like Bart's dad is touching himself*. This led researchers to question whether children with ASD are able to compute the hierarchical structural relationship of c-command, and raised the possibility that the children rely on a linear strategy for reference assignment. The current study investigates the status of c-command in children with ASD by testing their interpretation of sentences like (1) and (2) that tease apart use of c-command and a linear strategy for reference assignment.

1. The girl who stayed up late will not get a dime or a jewel (C-command)
2. The girl who didn't go to sleep will get a dime or a jewel (Non C-command)

These examples both contain negation (*not* or did*n't*) and disjunction (*or*). In (1), negation c-commands the disjunction phrase, yielding a conjunctive entailment. This gives rise to the meaning that the girl who stayed up late won't get a dime and she won't get a jewel. In (2), negation is positioned inside a relative clause and it does not c-command disjunction. Therefore, no conjunctive entailment follows. Thus, (2) is true if the girl just gets a dime or just a jewel, or possibly both. If children with ASD lack c-command, then (1) will not give rise to a conjunctive entailment. In this case, children might rely on a linear strategy for reference assignment. Since negation precedes disjunction in both (1) and (2), they might be interpreted in a similar manner. Likewise, children who show knowledge of c-command should perform well on sentences governed by Principle A. These hypotheses were tested in experiments with 12 Australian children with HFA, aged 5;4 to 12;7, and 12 typically-developing controls, matched on non-verbal IQ. There was no significant difference in the pattern of responses by children with HFA and the control children on either (1) and (2) or the Principle A sentences. The findings provide preliminary support for the proposal that knowledge of c-command and Principle A is intact in HFA children.

## Introduction

Individuals with ASD are known to have difficulties with language and communication. They present with little functional communication at one end of the spectrum to relatively well-developed language skills at the other (American Psychiatric Association, 2000). Nevertheless, no matter how proficient their language skills, all individuals diagnosed with autism share impairments in everyday use of language. Difficulties with pragmatics and prosody are understood as defining universal features of the disorder (e.g., Paul et al., 2005) but the status of grammatical development is less clear^1^. Some researchers have argued that grammatical knowledge is simply delayed in nature^2^ (Tager-Flusberg, 1981; Lord and Paul, 1997) while others argue that there are aspects of grammatical knowledge that are deficient (Pierce and Bartolucci, 1977; Bartolucci et al., 1980; Perovic et al., 2013a,b).

There have been few studies on complex syntactic structure in children with autism and there is not yet consensus on whether or not aspects of syntax are impaired. The issue is complicated by the range of abilities associated with ASD. Those who are classified as high-functioning (HFA) or score at least 70 on tests of non-verbal IQ^3^ (e.g., Howlin, 2003) tend to show sophisticated grammatical knowledge. One area of weakness that has been noted for children with low non-verbal IQ scores is morphosyntax. In particular, difficulties were observed for children's production of grammatical morphemes that mark “tense” (Roberts et al., 2004). The finding is that children with ASD tend to perform worse than children diagnosed with Specific Language Impairment (SLI). More recently, there have been investigations into the comprehension of complex syntactic structures such as *wh*-questions (Zebib et al., 2013), relative clauses (Riches et al., 2010; Durrleman and Zufferey, 2013; Durrleman et al., 2015), raising and passives (Perovic et al., 2007). Durrleman et al. (2016) assessed the comprehension of both relative clauses and *wh*-questions in French and showed that children with ASD had lower performance even on simple structures as compared with their typically-developing (TD) peers who were matched on non-verbal abilities^4^. Riches et al. (2010) showed that English-speaking teenagers diagnosed with autism and concomitant language deficits made significantly more errors than their age matched TD counterparts on subject and object relative clauses when tested on a sentence repetition task. A similar difficulty was also reported for the comprehension of relative clauses in French-speaking adults diagnosed with HFA (Durrleman and Zufferey, 2013; Durrleman et al., 2015). In an elicitation task for *wh*-questions, it was reported that French-speaking children diagnosed with autism avoided fronting in their *wh*-questions (Zebib et al., 2013). Importantly, these studies all involved movement or structures that encompass relations where the position that a phrase is interpreted differs from the position that the phrase is pronounced, a claim that parallels claims made for SLI (e.g., van der Lely and Pinker, 2014). Two recent studies by Perovic et al. (2013a,b) investigated the syntactic relation of binding.^5^ Binding does not involve movement but involves a dependency between two noun phrases. These studies are the impetus for our investigation on c-command in children with autism, so we introduce these in detail. Since the hierarchical relationship of c-command forms the basis for our experiments, we begin by introducing this abstract notion.

**Figure 1:**
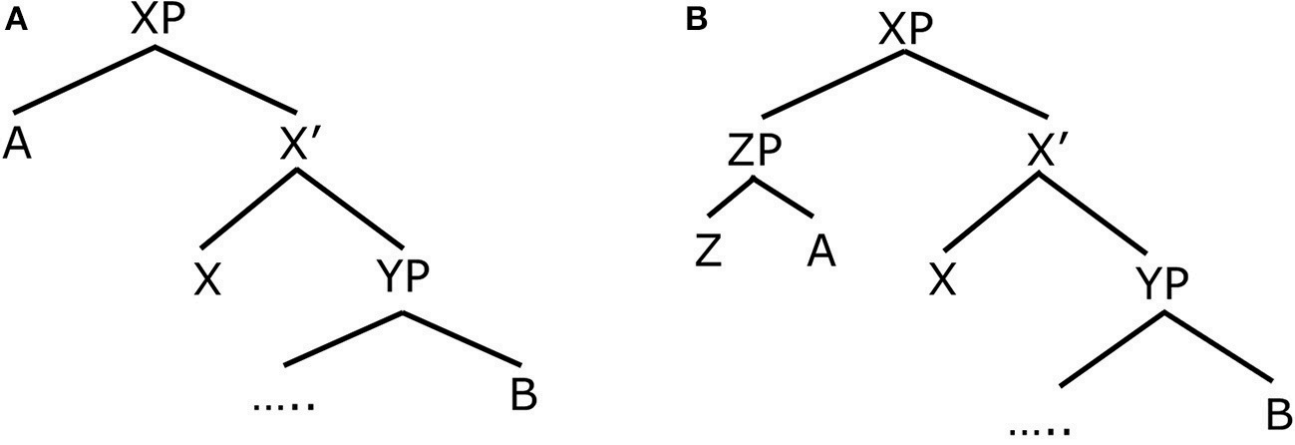
**(A)** Hierarchical Structure 1. **(B)** Hierarchical Structure 2.

C-command is a relationship between nodes in the phrase structure representation of a sentence. A node A is said to c-command a node B, if and only if the node that immediately dominates A also dominates B (see Koeneman and Zeijlstra, 2017). This is illustrated in Figure 1A. In this figure, the node that immediately dominates A is XP, because it is one step above A in the phrase structure. The node XP dominates B simply because it is higher than B in the tree, and it is possible to trace a path down the tree from XP to B. Therefore, A c-commands B. In Figure 1B, however, A does not c-command B. For A to c-command B, the node immediately dominating A would also have to dominate B. But the node immediately dominating A is ZP, and this node does not dominate B. This is because it is not possible to trace a path from ZP directly down the tree to reach B.

The Perovic et al. (2013a) study was based on an experiment conducted by Wexler and Chien (1985) that was designed to test children aged 2;6 to 6;6 years of age. The task was a two-picture Truth Value Judgement Task. In the original task, children were tested on sentences like *Cinderella's sister points to herself/her*, in which the subject noun phrase, *Cinderella's sister*, is a possessive noun phrase. This complex noun phrase provided the potential antecedents for *herself/her*. The child's task was to point to the picture that matched the sentence spoken by the experimenter. In one picture, Cinderella's sister was pointing to herself, and in the other, Cinderella was pointing to herself. The finding was that by 5 years of age, children were able to choose the correct referent for the reflexive 90% of the time.

The experimental stimuli used by Perovic et al. (2013a) used the same possessive noun phrase subjects but their stimuli featured the Simpson family. The stimuli included four different kinds of sentences, shown in (1) to (4) below.

1. Bart's dad is touching himself. Name Reflexive (NR)
2. Bart's dad is touching him. Name Pronoun (NP)
3. Bart's dad is licking a lamp post. Control Possessive (CP)
4. Bart is pointing to Dad. Control Name (CN)

Possessive noun phrases “*Bart's dad*” were chosen as the subject noun phrase because they allow for two potential referents (*Bart's dad* and *Bart*) for the reflexive or pronoun. This gives the child a choice between a c-commanding referent (*Bart's dad*) and a non c-commanding referent (*Bart*).

**Figure 2:**
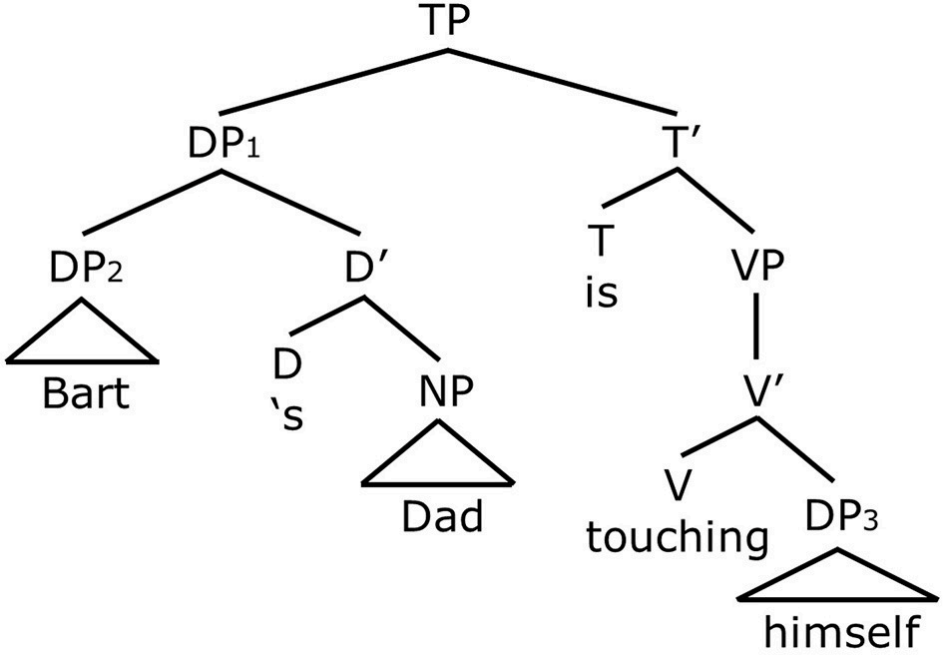
**The structure for *Bart's dad is touching himself***.

First let us consider how Principle A is satisfied in the sentences with a possessive noun phrase subject, like *Bart's dad is touching himself*. Intuitively, we know that *Bart's dad* is the only legitimate antecedent for *himself*, and that the other potential antecedent *Bart* is not, but let us verify this using the notion of c-command. Consider Figure 2. In Figure 2, *Bart's dad* is the subject of the sentence. This complex possessive phrase is represented by a Determiner Phrase (DP_1_). DP_1_ is broken down into further components—DP_2_ (*Bart*) and D' containing the possessive marker and the Noun Phrase, *Dad*. Applying the definition of c-command, DP_1_,*Bart's dad*, c-commands *himself* as the node immediately dominating DP_1_,the TP, also dominates DP_3_ which is the reflexive, *himself*. Now let us consider *Bart*. We see, the node that immediately dominates DP_2_ or *Bart* (that is DP_1_) does not dominate *himself*. Therefore, *Bart* does not c-command the reflexive and despite being in the same clause, it is not a potential antecedent.

A control condition with the same possessive subject noun phrase and no pronoun or reflexive in the predicate, as in (3), was included in order to test whether children knew the structure of possessive noun phrases and could distinguish between the two potential antecedents *Bart's dad* and *Bart* in sentences that were not related to knowledge of pronouns or reflexives. This condition also tested the c-command relation independently of binding^6^ in the sense that if children are able to compute the subject-predicate relations correctly then they should be sensitive to c-command.

The Perovic et al. (2013a) study tested 14 children diagnosed with autism, ranging in age from 6 to 17 years (*M* = 11;6). Twenty seven TD children aged 3–9 years matched on the Kaufman Brief Intelligence Test (KBIT) (KBIT-TD) and the Test for Reception of Grammar (TROG) (TROG-TD) formed the control groups. For the autism group, the mean standard score (SS) for the matrices subtest of the KBIT, a standardized test assessing non-verbal IQ, was 65.93. The mean for the TROG, a standardized test that assesses grammatical comprehension, was 56.5. The task was the same 2-choice picture selection task. The experimental findings revealed poor performance on sentences containing a reflexive as compared with ones containing a pronoun. The autism group had a mean correct of 67% on the sentences containing a pronoun (NP), while the two control groups, KBIT-TD and TROG-TD both scored a mean of 71% correct. On the sentences containing reflexives, the children with autism performed below chance, with a mean of 43% correct. This was significantly different from the control groups; the KBIT-TD group scored a mean of 92% correct and the TROG-TD group was 83% correct. There was some individual variation, however, with 2 of the 14 participants showing the pattern of better performance on reflexives rather than pronouns. On the control items containing a name (CN), the children with autism were significantly worse than the KBIT-TD group but not the TROG-TD group. The TD matched children tended to show better performance on sentences containing reflexives as early as age 5. Their performance was in accord with the patterns established in the previous literature (e.g., Wexler and Chien, 1985; Chien and Wexler, 1990)^7^.

Perovic et al. (2013a) interpreted the experimental findings to suggest that children with ASD have a syntactic deficit over and above any well-established pragmatic difficulties that are part of the disorder. First, because ASD children did not do well on reflexives, the authors interpreted this to mean that Principle A was either lacking or deficient in some way, as this pattern of better performance on pronouns than reflexives is not seen in TD children. They consider the proposal that children are assigning reference using a linear strategy to determine the antecedent for the reflexive, but leave this possibility open. Reliance on a linear strategy would mean that a child with autism could assume that an antecedent for reflexive is a preceding noun phrase that appears in the same clause as the reflexive. Such an assumption would lead to good performance on simple sentences like *Mary points to herself* but is expected to give way to poor performance on sentences like *Bart's dad is pointing to himself*. In this case, since there are two potential antecedents in the clause, so a child would end up guessing and choosing either *Bart's dad* or *Bart* as the antecedent. As c-command is needed to establish the relationship between the reflexive and its antecedent, Perovic et al. (2013a) interpreted this to mean that “children with autism do not show sensitivity to c-command in establishing the complex syntactic dependency of binding, where the antecedent of a reflexive must c-command the reflexive” (Perovic et al., 2013a, p. 25).

Not all studies have shown poor performance on reflexives. This was not the case for a study by Terzi et al. (2014) that compared performance of reflexives and pronouns in Greek-speaking children. Before we report the results, a little background on Greek is in order. Greek differs from English in that it has two kinds of object pronouns; strong pronoun and clitic pronouns. English is considered just to have strong pronouns. The strong pronouns in Greek differ from clitic pronouns in several ways. First, strong pronouns carry lexical stress which is not the case for clitics. Second, clitic pronouns can attach to the verb. Both kinds of pronouns also share important features. They both inflect for gender, number and case, and they are never used to refer to an antecedent that appears within the same clause. Turning to reflexives, Greek reflexive pronouns are subject to Principle A just like English reflexives, and the antecedent for a Greek reflexive must appear in the same clause as the reflexive. Previous research has shown that Greek-speaking TD children master use of both strong and clitic pronouns at an early age (see Varlokosta, 2000). With this background in place we can turn to Terzi et al.'s (2014) study. In this study, the children with autism were classified as high functioning based on their high non-verbal IQ (>80). The control group consisted of TD children individually matched to the participants with autism based on raw scores of a vocabulary test. The experiment showed that the children with autism performed worse than TD children only on clitics or clitic pronouns (88.3% correct) in contrast to reflexive pronouns (97.5% correct) and strong pronouns (94.9% correct). The Greek children with autism performed better than the English-speaking children in Perovic et al.'s experiment, where they were only 43% correct on sentences containing reflexives. However, it is important to acknowledge that there are slight differences between Greek reflexive pronouns and English ones. Greek reflexives are complex forms and are inflected for case and number. Most importantly, reflexivity is not just expressed through reflexive pronouns but it is also expressed through special verbal morphology. The results from the Greek-speaking children are more in line with recent results obtained for 26 British HFA children who showed good comprehension of reflexives (Janke and Perovic, 2015) on a two-choice picture selection task.

In a later study, Perovic et al. (2013b) re-assessed their experimental findings, with a larger group of children with autism, aged 6–18 years, using the same stimuli given in (1)–(4). In this study, participants were divided into two subgroups according to the presence or absence of language impairment as measured by their scores on tests of both receptive language (TROG-2; PPVT-3) and productive language (a vocabulary subtest of the KBIT). The group with language impairment, the ALI group, consisted of participants scoring below the 10th percentile on at least 2 of the three tests. In this experiment, only the ALI group performed worse on the sentences containing reflexives (*M* = 49% correct) such as (1) as compared to the sentences containing pronouns (*M* = 71% correct) like (2). The ALI group consistently showed chance performance on sentences containing reflexives. Thus, this group of children did not seem to distinguish between potential antecedents (*Bart's dad* and *Bart*) for the reflexive. The performance of the ALI group was not different between the Name Pronoun (NP) and other control conditions, the Control Possessive (CP) condition (*Bart's dad is licking a lamp post*; *M* = 77% correct) and the Control Name (CN) condition (*Bart is pointing to Dad*; *M* = 79% correct). The performance of the children without language impairment, the ALN group, showed better performance on sentences containing reflexives (*M* = 96% correct). Both the ALI and the ALN groups (*M* = 83% correct) showed delayed comprehension of pronouns consistent with a delay in their “linguistic” pragmatic knowledge.

The results from the larger group of children with autism provided support for the proposal that Principle A is either missing or incorrectly represented in the group of children designated as ALI. The authors elaborate their proposal as follows: “It is not necessarily the case that children with ALI cannot compute c-command; they might be able to use it to constrain representations in other constructions” (Perovic et al., 2013b, p. 146). The authors further propose that the “ALI version of Principle A constrains the ALI child only to having a clause-mate antecedent of the reflexive, missing the c-command part of Principle A” (Perovic et al., 2013b, p. 146).

However, one could ask that why is c-command missing only in the application of Principle A, given that it is a very general hierarchical notion. The puzzle is that the ALI group performed quite well on the Control Possessive condition (CP) that was hypothesized to test for c-command relations outside the domain of binding. On these trials, the children were 77% accurate. So, we might ask why children performed poorly on the sentences containing reflexives (49%), but well on the controls, given that c-command is necessary to identify the correct antecedent in both cases. To explain this puzzle, we could interpret Perovic et al.'s discussion in the following way. In order to pick out the correct antecedent for the reflexive, children not only need to have knowledge of c-command, but they also need to understand that the relationship between the reflexive and its antecedent is one of variable binding^8^. Given the added complexity introduced by variable binding, children end up guessing, hence their chance performance. In the control sentences such as *Bart's dad is licking a lamppost*, c-command is still a necessary prerequisite for identifying the correct referent. In this case, without the added complexity of variable binding, children are able to use a linear strategy to identify a clause-mate antecedent. This strategy means they tend to take the nearest noun as the referent for the antecedent, so they choose the noun *dad* from *Bart's dad*, and overall, end up with roughly 77% correct performance on the CP control items^9^.

Other issues arise when considering the difference in results between the ALI and ALN groups of children (Perovic et al., 2013b). The ALI children examined by Perovic et al. (2013b) had low non-verbal abilities. This observation suggests that children on the lower end of the autism spectrum have problems with advanced syntactic structures and serves as motivation for matching TD and ASD children in terms of non-verbal abilities. In our study, we chose to examine children on the higher end of the spectrum (see Terzi et al., 2016a,b). One question that arises is why the ALN children performed well in the second study by Perovic et al. (2013b). Is it the case that this group of children has c-command in place, in contrast to the ALI children? Or, is it the case that due to their high non-verbal abilities and superior language skills, they were able to adopt a linear strategy for both the variable-binding sentences containing reflexives and the control sentences? Or, did children use c-command to identify the appropriate subject noun phrase for both the Name Reflexive sentences and the Control Possessive sentences? The sentences with a possessive NP (e.g., *Bart's dad*) used in this study do not allow us to tease apart the difference between using c-command and using a linear strategy to correctly identify the antecedent, so we turn to a different structure to further investigate these possibilities in children with ASD.

We introduce a novel experiment that differentiates between interpretations computed based on the basis of knowledge of c-command and ones based on linear order. The experiment rests on the interpretation of disjunction (“or”). There has been some debate in the literature over whether “or” in child language corresponds to “inclusive-or” as in classical logic, or “exclusive-or,” so we review this briefly here. Some researchers have pointed out that the majority of input to children is consistent with “exclusive-or” because the contexts in which children hear disjunction are ones in which only one of the disjuncts is true (Braine and Rumain, 1981, 1983; Morris, 2008). For example, Morris (2008) examined 240 transcripts of parent-child interaction in the CHILDES database (MacWhinney, 2000). There were 465 spontaneous uses of “or” in a corpus of 100,626 conversational turns. In this corpus, “or” was used in situations where only one of the disjuncts was true between 75 and 95% of the time. As Crain and Khlentzos (2007, 2010) point out, however, a situation in which one disjunct is true is also consistent with “inclusive-or.” If “or” is “inclusive-or,” then A or B is true when A is true; when B is true or when A and B is true. So, the fact that children hear disjunction in a context in which one disjunct is true does not favor the proposal that children understand disjunction as “exclusive-or.”

Consider a statement such as “*Every child took a tiger or a dinosaur*.” This is true in circumstances in which there are three children; one takes a tiger, one takes a dinosaur and the third child takes both a tiger and a dinosaur. Adults reject such a statement in this circumstance, however. This is argued to be due to the implicature of exclusivity. Children, on the other hand, have been found to be less sensitive to the implicature. For example, Gualmini (2003) tested children's interpretation of sentences like *Every child took a tiger or a dinosaur* in the story context just described, in which one child chooses a tiger, another a dinosaur, and the third child chose both animals. Unlike adults, the child participants accepted the puppet's description 71% of the time. In certain contexts, such as contexts of betting or uncertainty, however, the implicature of exclusivity is canceled, and then the finding is that adults, too, accept sentences with “or” in all three circumstances. In a further experiment using conditional sentences such as “*If a giraffe or a penguin is on the stage, then I get a coin*,” Gualmini et al. (2001) established a context of uncertainty, and showed that in this case, also, children assign the range of truth conditions consistent with “inclusive -or.” In this experiment, certain animals, such as a giraffe, or a penguin or both, were placed on the stage, and then the stage curtains were opened to reveal the animal or animals. The puppet produced the conditional statement before the curtains were opened. This way, there was uncertainty about the outcome. Once the curtains were opened, the puppet asked if he got a coin. On some trials, disjunction “or” was replaced with conjunction “and.” Children clearly distinguished the truth conditions of disjunction vs. conjunction. When conjunction was used, as in “*If a giraffe and a penguin are on the stage, then I get a coin*” children rejected the sentence when only one animal was on the stage, unlike when disjunction was used in the sentence. These experimental findings suggest that “or” is interpreted as “inclusive-or” in child grammar.

Crain (2008) points out that the “exclusive-or” interpretation of disjunction yields different properties in negative sentences. Recall that “A or B” is true only if exactly one of the disjuncts, A or B, is true. It follows that sentences of the form “Not A or B” are false only if exactly one of the disjuncts, A or B, is true. Sentences of the form “Not A or B” are true, therefore, if both disjuncts are true, and they are also true if both are false. The fact that sentences of the form “Not A or B” are true if both disjuncts are true is a consequence of interpreting “or” as “exclusive-or,” (see Crain et al., 2000). Suppose that John says the following “*Mary did not bring ice cream or cake to the party*.” If John's use of disjunction is interpreted as “exclusive-or,” then his assertion would be true if Mary brought both ice cream and cake to the party, which is clearly not how native speakers of English interpret this sentence.

**Figure 3:**
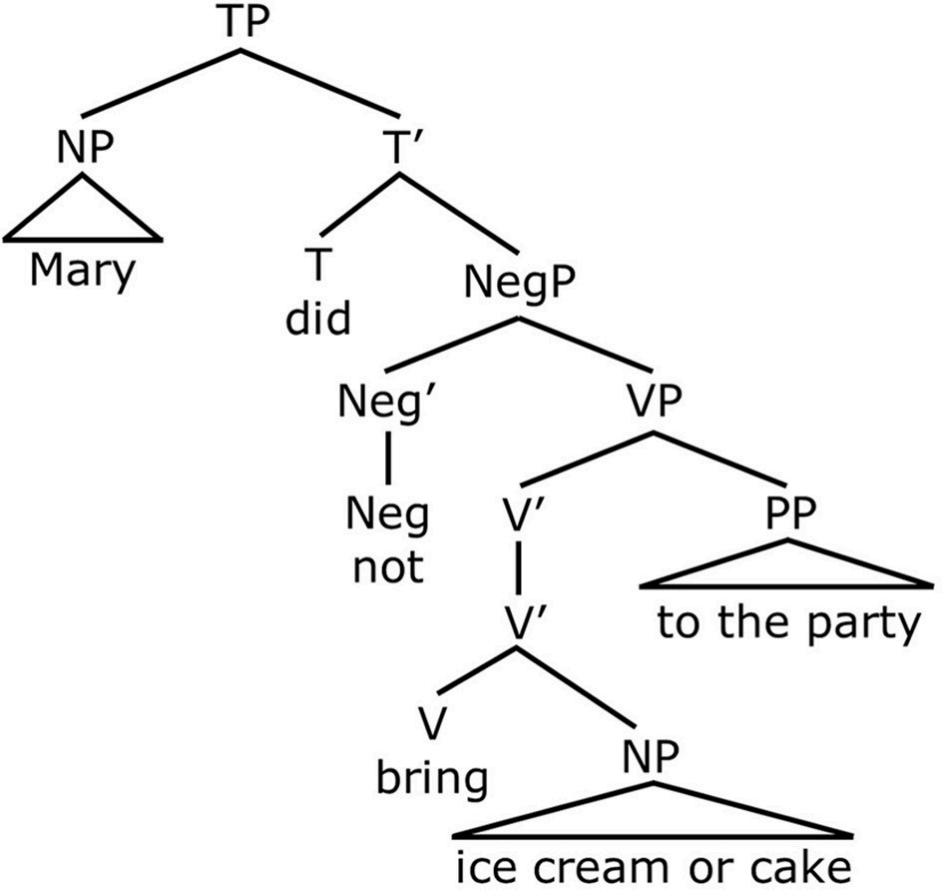
**Representation for *Mary did not bring ice cream or cake to the party***.

On the other hand, if disjunction is “inclusive-or,” then John's statement “*Mary did not bring ice cream or cake to the party*” is only true in circumstances in which Mary brought neither dessert to the party. Intuitively, this is the right result for English. The meaning of such sentences containing negation and disjunction corresponds to one of the laws of propositional logic, according to which a negated disjunction “Not (A or B)” logically entails the negation of both disjuncts. It entails “Not A” and it entails “Not B.” In classical logic, this law is stated in one of De Morgan's laws: ¬ (A ∨ B) => (¬A ∧ ¬B). The interpretation that is captured by De Morgan's law depends on the interpretation of disjunction as being within the scope of negation, where scope assignment corresponds to the structural notion of c-command in linguistic theory (see Crain, 2012). This can be seen in Figure 3, where *not* c-commands disjunction, *or*, which is contained in the object noun phrase. In other words, a “conjunctive” entailment is licensed by disjunction in the scope of negation, just as long as disjunction is analyzed as “inclusive-or” (Crain, 2008). From this point, we will simply assume that “or” is interpreted as “inclusive-or” in child grammars.

Children's interpretations of sentences containing negation and disjunction were tested in a study by Crain et al. (2002) in typically-developing children aged 3;11- to 5;9 years. This study is the basis for our study with a group of children with ASD, so we review it in some detail. The relevant sentences from the Crain et al. (2002) study are given in (5) and (6).

(5) The girl who stayed up late will not get a dime or a jewel
(6) The girl who didn't go to sleep will get a dime or a jewel

**Figure 4:**
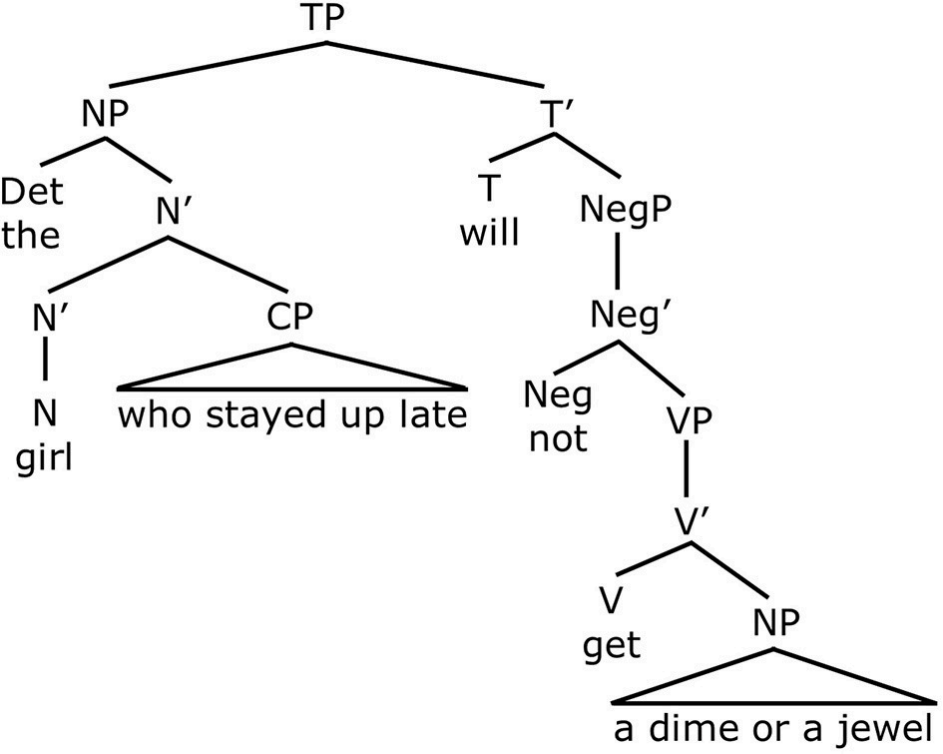
**Negation c-commands Disjunction**.

**Figure 5:**
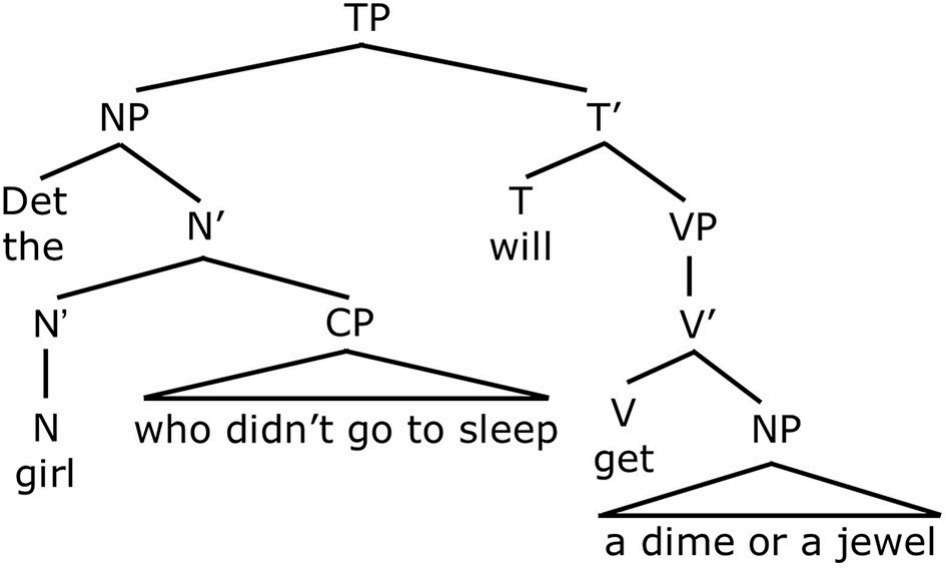
**Negation does not c-command Disjunction**.

Notice that both of these sentences contain negation (either *not* or *n't*) as well as the disjunction word *or*. However, they yield different interpretations. In (5), negation is in the main clause and c-commands disjunction. See Figure 4. As noted above, when disjunction is in the scope of negation, this gives rise to the conjunctive entailment. That is, (5) means that the girl who stayed up late will not get a dime AND the girl who stayed up late will not get a jewel. This is the only available interpretation for this sentence. In the sentence in (6), as in (Figure 5), negation precedes disjunction. In this case, however, negation, which is part of the negative auxiliary verb *didn't*, does not c-command disjunction. This is because *didn't* is embedded inside the relative clause *who didn't go to sleep*, that modifies the subject noun phrase. Therefore negation does not c-command disjunction in Figure 5. Because negation does not c-command disjunction, the sentence does not give rise to a conjunctive entailment. Rather, it gives rise to disjunctive truth conditions. This means that the sentence means the girl who didn't go to sleep will get a dime, or the girl who didn't go to sleep will get a jewel (or possibly both). Therefore, Crain et al.'s (2002) predictions were as follows. If children have knowledge of c-command, they will generate the conjunctive entailment for (5) and reject the sentence. On the other hand, they should accept (6), since there is no c-command relation between negation and disjunction in this sentence. If children do not have c-command, however, and rely on a linear strategy to interpret sentences containing negation and disjunction, they should treat the two sentences in the same way. In this case, it is likely that children will not enforce the conjunctive entailment but attribute disjunctive truth conditions to both sentences.

The experiment conducted by Crain et al. (2002) used the Truth Value Judgement Task (TVJT) to test sentences like (5) and (6) (Crain and Thornton, 1998). Thirty children, ranging in age between 3;11 and 5;9 (mean age of 5;0), participated in the experiment. The experiment was conducted over two sessions. The participants were also divided in two groups. One group was presented with two trials of sentences like (5) in session 1 and two trials of sentences like (6) in session 2. The other group heard the target sentences in the opposite order. The child participants watched a story acted-out by one experimenter along with a puppet, played by a second experimenter. At the end of the story, the puppet described what he thought happened in the story. The child's task was to judge whether or not the puppet said “the right thing.” That is, children judged the truth or falsity of the puppet's description of the story. To ensure that use of disjunction was felicitous in the experimental context, the stories were presented in “Prediction Mode.” Instead of having children judge the truth of the puppet's statement at the end of the story, the puppet made a prediction about forthcoming events at some point in the middle of a story (and it was repeated again at the end). Thus for (6), for example, half-way through the story, the puppet predicted how the events might unfold by uttering the target sentence, *The girl who doesn't go to sleep will get a dime or a jewel*. In such contexts of uncertainty, it is felicitous to use disjunction. If the child judged the puppet's statement to be true, then it was assumed that the child's grammar generated a structure and a meaning for the sentence that matched the events that took place in the story. If the child judged the puppet's statement to be false, then it was assumed that the child's grammar generates only structures and meanings that did not match the events in the story. This inference is based on the assumption that, whenever possible, children (and adults) will access a meaning that makes the puppet's sentence true. This is called the Principle of Charity (Davidson, 2001). If children and adults adhere to the Principle of Charity, then we are invited to make the following inference: When children and/or adults consistently judge a sentence to be false, this indicates that they were unable to mentally compute a meaning representation that makes the sentence true.

The story that was used to test the sentences in (5) and (6) acted-out a tale of two girls who were waiting for the tooth fairy to arrive, as they had both lost a tooth. The girls knew that the tooth fairy would come during the course of the night and give them a reward in exchange for their lost tooth. One of the girls decided to go to sleep, as all good girls should do, but the other girl decided to stay awake. The tooth fairy duly arrived, bringing along two dimes and two jewels. At this juncture, the puppet made his prediction about what would happen next. This was the puppet's delivery of the test sentence. The story then resumed. The fairy gave both a jewel and a dime to the girl who was sleeping. The girl who was awake explained that she knew she should be asleep but had stayed up because she really wanted to meet the tooth fairy. The tooth fairy said that she was disappointed that the girl had not gone to sleep, but still decided to give her one reward. She gave the girl just a jewel. At the end of the story, the puppet repeated the prediction made in the middle of the story, delivering the test sentence for the children to judge.

The main finding from the experiment was that children rejected sentences like (5), in which negation c-commanded disjunction 92% of the time. That is, children rejected the sentences because they generated the conjunctive entailment. They took (5) to mean that the girl who stayed up late didn't get a dime and she didn't get a jewel. This is false, because in the story the tooth fairy gave her a jewel. Sentences like (6) were accepted 87% of the time. There was no c-command relation between negation and disjunction in (6). The children generated disjunctive truth conditions; they accepted a sentence like (6) because it was true that the girl who didn't go to sleep got a dime or she got a jewel. The Crain et al. experiment showed that typically-developing children treat the two sentence types very differently, rejecting one, and accepting the other. This suggests that children are generating hierarchical sentence representations, and that the notion of c-command guides their interpretation of these sentences. As noted, if children had been relying on linear precedence or a linear strategy, then there would be no reason to generate a conjunctive entailment for sentences like (5). Thus, it is likely that both sentences would have been interpreted in a similar manner with disjunctive truth conditions.

Returning to children with autism, recall that the Perovic et al. (2013b) experimental findings showed that the ALN group of children did well on Principle A, correctly identifying the appropriate referent 96% of the time. However, given that the correct referent (*Bart's dad* not *Bart*) both c-commands the reflexive, and is, from a linear perspective, the closest and perhaps most salient potential antecedent, it was difficult to know whether the children were drawing on grammatical knowledge or a linear strategy. For this reason, we replicate the Crain et al. (2002) study which distinguishes an interpretation based on c-command from one based on a linear strategy.

## Experiment 1: disjunction and negation

The goal of the experiment is to determine how children with ASD interpret sentences containing negation and disjunction. Crucially, this structure disentangles the confound present in the Principle A structure used in Perovic et al. (2013a,b). In sentences with reflexives containing possessive noun phrase subjects like *Bart's dad*, it was not possible to tell whether correct identification of the antecedent for the reflexive could be attributed to knowledge of c-command or a linear strategy. The comparison structure with negation and disjunction tested in the present study dispenses with this confound. In addition, our second experiment also incorporates the sentences containing reflexives as used by Perovic et al. for comparison.

The first experiment used sentences with the same structure as the study by Crain et al. (2002), ones *like* (7) and (8). In (7) negation c-commands disjunction, while in (8), it does not because the negative auxiliary verb is embedded inside the relative clause.

(7) The boy who is on the bridge will not get a ball or a car
(8) The boy who isn't on the bridge will get a ball or a car

The experimental hypothesis was as follows: If children with ASD can access c-command, they should respond to such sentences in the same way as typically-developing children. That is, children should interpret (7) as the boy who is on the bridge will not get a car and the boy who is on the bridge will not get a ball. In other words, they should generate a conjunctive entailment when disjunction appears in the scope of negation. However, if children with ASD cannot access the requisite notion of c-command, they should draw no distinction in the interpretations assigned to sentences (7) and (8). In this case, they would be unlikely to enforce a conjunctive entailment for (7). Furthermore, if c-command is not guiding children's interpretations, they may adopt a linear strategy. Since negation precedes disjunction in both (7) and (8), the expectation is that children would interpret both sentences in the same way, with disjunctive truth conditions.

### Methods

#### Participants

Twelve children on the autism spectrum participated in the study. Their age ranged from 5;4 to 12;7, with a mean of 9;11 years. Children with autism were recruited from a special school for children with ASD, located in Melbourne. Children in Sydney were recruited from a Special Education Centre. In addition to these schools, children diagnosed with autism were also recruited from advertisements placed on the Autism Spectrum Australia (ASPECT) website. A formal diagnosis of autism was established based on previous assessment reports as provided by the parents or children and as identified by the specialist school. The children who made up the control group (typically-developing children) were recruited from general advertisements placed on Macquarie University campus. Only children whose first language was English were recruited for both the groups. Ten adults were also recruited in the pilot phase for the experiment. They were students recruited from general advertisements across the campus. This study was carried out with the approval of Human Research Ethics Committee at Macquarie University (Ref: 5201200880).

The children with autism all had verbal communication skills. This group of children was tested on standardized tests of language and cognition. These tests included the matrices subtest of KBIT (Kaufman and Kaufman, 2004) measuring non-verbal IQ and the Test for Reception of Grammar Second Edition (TROG-2; Bishop, 2003). Based on the scores of the KBIT, the children who formed the group with ASD can be described as high-functioning (HFA), as the majority of children had a standard score of more than 80 (Howlin, 2003; Norbury, 2005). The TD children (*n* = 12) were matched to the children with autism within 2 points of the KBIT raw scores. The age of the children in the matched comparison group ranged from 5;10 to 8;10, *M* = 7;1. Table 1 summarizes the descriptive data.

**Table 1:** **Participants' ages and mean scores (standard deviations) on standardized tests of language and cognition**.

	**Autism (*n* = 12)**	**TD (*n* = 12)**
Mean chronological age in years (SD)	9;11 (2;4)	7;1 (0;9)
Range	5;4 – 12;7	5;10–8;10
KBIT matrices standard scores (SD)	94.41 (12.19)	114.8 (11.10)
Range	74-121	91–127
KBIT matrices raw scores (SD)	25.75 (6.19)	25.25 (6.07)
Range	15–34	16–34
TROG 2 Raw scores (SD)	9.67 (5.06)	–
Range	3–16	–
TROG 2 Standard scores (SD)	76.58 (17.05)	–
Range	55–104	–

#### Procedure

**Figure 6:**
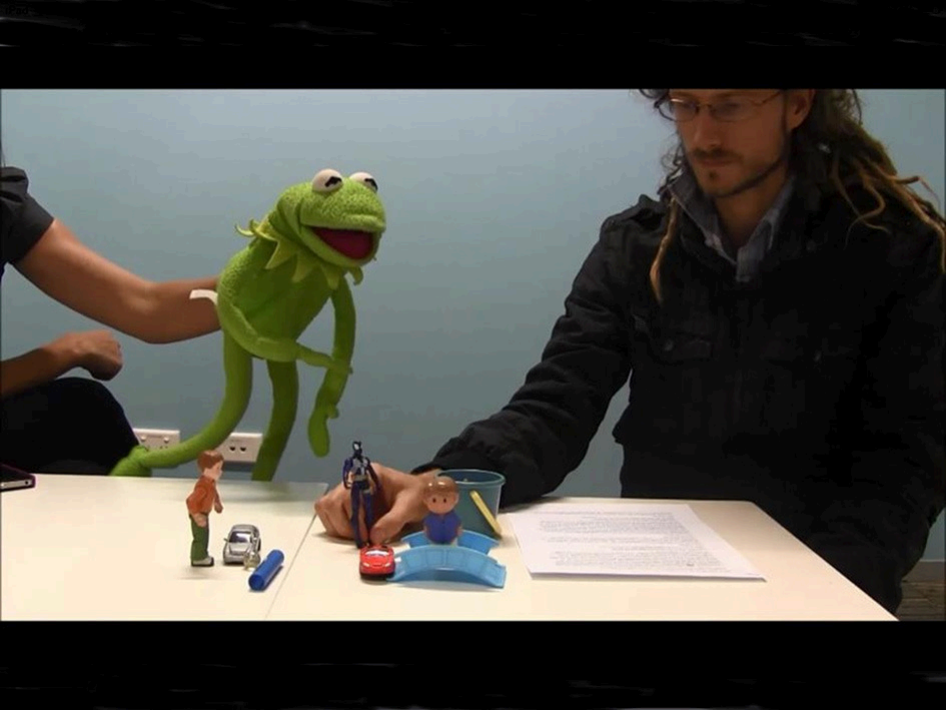
**A trial from Experiment 1**.

Before testing, all caregivers provided informed consent for their child's participation, in accordance with ethical guidelines set out by the Human Research Ethics Committee at Macquarie University. The present experiment used the dynamic TVJT (Crain and Thornton, 1998) as in the study by Crain et al. (2002). The task of the child was to judge the truth or the falsity of the given sentence spoken by the puppet. In order to make disjunction felicitous in the context, the TVJT was adapted to use in the prediction mode (see Chierchia et al., 1998), again, as in Crain et al. (2002). Accordingly, our story was interrupted half way through and the first experimenter who acted as a dog while manipulating toys asked Kermit, the second experimenter, what he thought would happen next. Kermit replied by uttering the target sentence and the story resumed. At the end of the story, Kermit repeated his prediction to remind the children about the events that occurred in the story. In the present experiment, the stories were videotaped, and the videotaped scenarios were presented to the children on an iPad. This step ensured consistent presentation, and allowed a single experimenter to present the stimuli. The experimenter who demonstrated the iPad videos to children instructed them to judge Kermit's sentences at the end of each story. The trials in our study were not split up into different sessions as was done by Crain and colleagues. All the participants in our study heard all the test trials in the same session. See Figure 6 for a snapshot of the experimental trial.

Each child was tested individually either in a quiet corner of a room at the school or in the Language Acquisition Lab at the University. The testing for each child, including the standardized tests lasted for approximately 1.5 hours. If the child had difficulty paying attention, the session was split into two parts. All participants were told that they would watch short stories and hear a puppet who tries to say what would happen next. Their task would be to evaluate whether the puppet in the iPad presentation was right or wrong. If the puppet was wrong, children were asked why they thought that the puppet was wrong. All the verbal responses of children were digitally recorded. Children's judgments of the test sentences were scored as “Yes” or “No.” Percentages of correct rejections or acceptance for particular items were calculated for each child.

#### Stimuli

The experiment included 4 stories to test the structure in (7) in which there is a c-command relation between negation and disjunction. The c-command sentences like (7) were associated with rejections of the test sentence, in keeping with the TVJT methodology. In order to demonstrate their knowledge of c-command and the resulting constraint on interpretation, children had to overcome the Principle of Charity and reject the sentences. There were also 4 stories for the kind of structure as exemplified in (8), in which there is no c-command relation between negation and disjunction. One of the four stories was associated with rejection in order to catch a biased style of responding where children might implicitly pair c-command stories with a “no” response and non c-command stories with a “yes” response. The stories were presented/played to each child in random order. The experimenter chose the video clip of any story at random for presentation. The testing session started with two practice trials. Both the practice trials were paired with a “no” response. These practice trials both contained negation (e.g., “*The cook will not let Elmo eat the cake*”). See Table 2 for a complete list of all test sentences.

**Table 2:** **List of sentences for C-command and Non C-command trials**.

**No**.	**C-command sentences**	**Correct response (character gets one of the objects mentioned)**
1	The boy who is on the bridge will not get a ball or a car	Reject
2	The cat who is on foot will not get a fish or milk	Reject
3	The Dino who is on the building will not get a potato chip or peanut	Reject
4	The Penguin who is on the barrel will not get a coin or a jewel	Reject
**No**.	**Non C-command sentences**	**Correct response**
1	The girl who is not on her bed will get cheese or salad	Reject
2	The mermaid who is not on the plant-island will get a crown or a seahorse	Accept
3	The thief who is not on the speed boat will get a blanket or tea	Accept
4	The gardener who is not on the barrel will get a hat or a seed-bottle	Accept

#### Results

The main finding was that the group of children with ASD performed in a similar manner to the typically-developing group of children, rejecting sentences like (7) and accepting ones like (8)^10^. The group results are summarized in Table 3.

**Table 3:** **Percentage of correct interpretations (Group Mean) for C-command and Non C-command sentences**.

**Sentence types**	**ASD(%)**	**TD(%)**	**Group difference**
C-command	89.6	100	Not significant
Non C-command	66.7	68.7	Not significant
Group difference	Significant	Significant	

When children were asked why they rejected the c-command sentences like (7), the children from both groups gave similar justifications. For example for the test sentence, *The boy who is on the bridge will not get a ball or a car*, the children would say that the puppet is wrong as the boy on the bridge got a car whereas he was not supposed to get anything. For another test sentence, *The cat who is on foot will not get a fish or milk*, the children would say that the puppet is wrong as the cat on foot got a fish. A Mann-Whitney Test showed there was no significant difference in the responses of the HFA group and the TD children for the c-command sentences (*Z* = 1.3568, *p* = 0.17384) or for the non c-command sentences (Z = 0.4907, *p* = 0.62414). A Mann-Whitney Test was also conducted to compare performance on the c-command and non c-command sentences within both the groups. The difference was significant for both the HFA (*Z* = 2.4537, *p* = 0.01428) and the TD groups (*Z* = 3.7816, *p* = 0.00016).

There was a significant difference in performance between the two types of sentences for both the groups. This finding is comparable to that obtained by Crain et al. (2002). In their experiment, children rejected the c-command sentences like (7) 92% of the time, and accepted the non c-command trials 87% of the time. The similar pattern suggests that children with HFA are able to use the hierarchical structure of c-command to distinguish between the c-command and the non c-command sentences just like their TD peers. We return to possible reasons for the fact, that children were not as accurate on the non c-command sentences as the c-command trials, in the Discussion section.

In the next experiment, we explore whether these same children are able to implement c-command to assign the correct referent for reflexives on sentences governed by Principle A. If children are able to use c-command to constrain relations between negation and disjunction, then it is conceivable that they may still show sensitivity to c-command in establishing the complex syntactic dependency of binding, unless variable binding is an issue. If children adopt a linear strategy in conditions where they face the added complexity of variable binding then their performance on the control sentences would be better than their performance on Principle A sentences.

## Experiment 2: Principle A

The present study is concerned with Principle A and reflexives. Previous studies conducted by Perovic and colleagues make it difficult to conclude whether the ALN children did well in identifying the correct referent for the reflexive in sentences like *Bart's dad is touching himself* because their grammatical knowledge incorporates the notion of c-command or whether they were simply adopting a linear strategy. As we saw, *Bart's dad* is the only legitimate antecedent for *himself*, and the other potential antecedent *Bart* is not a potential antecedent because it does not c-command *himself*.

### Methods

#### Participants

The same child participants (*n* = 12) who participated in Experiment 1 also participated in Experiment 2. Their age ranged from 5;4 to 12;7, with a mean of 9;11 years. Eight adults who did not participate in Experiment 1 participated in a pilot study to ensure the viability of the tasks. They were recruited from general advertisements across Macquarie University.

#### Procedure

**Figure 7:**
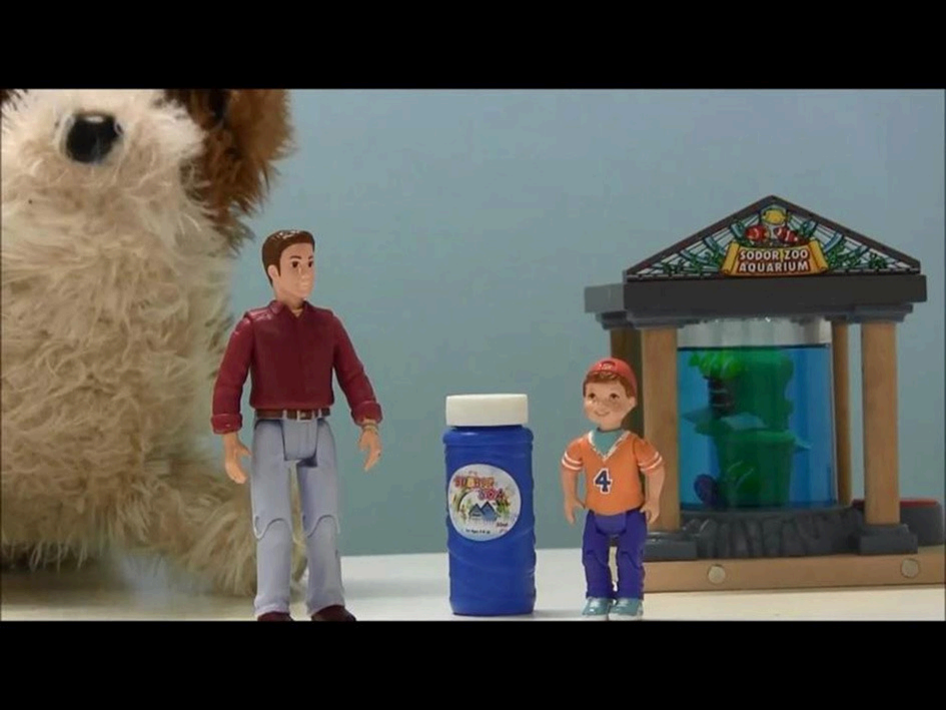
**A trial from Experiment 2**.

The children were tested on sentences containing reflexives that are governed by Principle A using the dynamic version of the Truth Value Judgment Task (TVJT) (Crain and Thornton, 1998). Our methodology contrasts with that of Perovic and colleagues who used a 2-choice picture selection task. In our experiment, as in Experiment 1, the stories were pre-recorded and presented to the children on an iPad. See Figure 7 for a snapshot of the experimental trial. The testing procedure was similar to Experiment 1 except that this experiment did not adopt the prediction mode. This experiment used the “description mode” in which the puppet simply tried to say what happened in the story on its completion. The experimental items were preceded by two practice items, one designed to be a “Yes” answer, and the other a “No” answer. The experimenter then proceeded to the main task. The first story presented to children was always a Name Reflexive (NR) story containing a reflexive like (1) followed by a Control Possessive structure story (CP) like (3). Children were presented with four stories under each category. At the completion of each story, children judged two sentence types; first they judged a Name Reflexive story, and then a Control Possessive story. Children's judgments of the test sentence were scored as “Yes” (true) or “No” (false).

#### Stimuli

The target sentences for the second experiment were sentences containing reflexives like *Bart's dad washed himself with soap*. This was the same structure as used in the Perovic et al. experiment, with the addition of a Prepositional Phrase (PP) such as *with soap* sentence-finally, to make the sentence seem more natural. The correct response associated with the Name Reflexive sentences was always a rejection of the test sentence. The target sentences were designed to be false to ensure that children have to override the Principle of Charity. In order to show their knowledge of the Principle A constraint, children have to go out of their way to reject the sentence in context, and to explain why it is false. In addition to the four Name Reflexive target sentences, there were four Control Possessive (CP) sentences; 2 of these were designed to be true and 2 were false. This was done in order to balance the “yes” and “no” responses. These also had the additional PP sentence finally (e.g., *Bart's dad washed the dog with shampoo*). See Table 4 for a complete list of test sentences.

**Table 4:** **List of sentences for Principle A**.

**Name reflexive (NR)**	**Control possessive (CP)**
Bart's dad washed himself with soap	Bart's dad washed the dog with shampoo.
Robot's master covered himself with a blanket	Robot's master poked the creature with a stick
Donald Duck's friend dressed himself in the costume.	Donald Duck's friend decorated the bird with the feather.
Spiderman's Brother dusted himself with the hairbrush	Spiderman's Brother cleaned the rock with the circus hat

#### Results

The main finding was that both the children with ASD and the TD control group all performed extremely well on the task. See Table 5 for group mean results of the children.

**Table 5:** **Percentage of correct interpretations (Group Mean) for NR and CP**.

**Sentence types**	**ASD(%)**	**TD(%)**	**Group difference**
Name Reflexive (NR)	79	94	Not significant
Control Possessive (CP)	85	83	Not significant
Group difference	Not significant	Not significant	

Each “No” response for the Name Reflexive test sentences was scored as correct rejection. Responses under the control possessive or CP condition were scored depending upon whether children correctly accepted or rejected the test sentences. A Mann-Whitney Test was used to compare the patterns of responses by children with autism and the TD children. The group difference (ASD vs. TD children) was not significant for the Name Reflexive sentences (*Z* = 1.0681, *p* = 0.28462) or for the Control Possessive sentences. (*Z* = 0.2887, *p* = 0.77182). Within-group analyses were also conducted across the two sentence types. The difference between NR and CP phrases were not significant for either the ASD (*Z* = 0.0289. *p* = 0.97606) or the TD control group (*Z* = 1.5588. *p* = 0.11876). When children were asked why they rejected the test items, children from both groups gave similar justifying responses. For example for the test sentence *Bart's dad washed himself with soap*. Children's stated reason for rejecting it was that *Bart's dad washed Bart with soap*.

In a nutshell, the performance of the HFA group does not differ across the Name Reflexive sentences and the Control Possessive structures. Children with HFA assigned the correct referent for reflexives on sentences governed by Principle A, just like their TD peer group.

## General discussion

Previous studies have shown that in contrast to high-functioning children with autism (HFA), children on the lower end of the autism spectrum and those with concomitant language impairments have difficulty in correctly interpreting sentences containing reflexives (Perovic et al., 2013a,b). Perovic et al. argued that the difficulty in interpreting reflexives when they appear in possessive structures like *Bart's dad is touching himself* arises because children may adopt a linear strategy, permitting both *Bart's dad* and *Bart* as potential clause-mate antecedents for the reflexive. As a result, children show chance performance. Thus, the authors argued that at least the “ALI version of Principle A constrains the ALI child only to having a clause-mate antecedent of the reflexive, missing the c-command part of Principle A” (Perovic et al., 2013b, p. 146). However, this hypothesis did not make it clear as to why the ALN children perform better on the Name Reflexive sentences and the Control Possessive sentences. Using the Principle A sentences with a possessive noun phrase antecedent like *Bart's dad*, it was not possible to tell whether these children were using their grammatical knowledge of c-command or a linear strategy to identify the correct antecedent. For this reason, we tested a new structure in which these two possibilities are differentiated, the sentences containing negation and disjunction, which we termed the c-command and non c-command sentences as illustrated in (7) and (8) respectively.

We hypothesized that if children with HFA have knowledge of the hierarchical relationship of c-command, they would generate a conjunctive entailment for the c-command sentences like *The boy who is on the bridge will not get a ball or a car* as disjunction appears within the scope of negation. That is, they would (only) get the interpretation on which the boy who is on the bridge will not get a ball and he will not get a car. However, if the grammatical knowledge of this HFA group of children was compromised, we predicted that the children would interpret the c-command and non c-command sentences in a similar manner. In this case, they would not be expected to impose a conjunctive entailment on sentences like (7). Presumably, in this case they would give the sentence the range of disjunctive truth conditions that arise for (8). That is, they would allow it to mean that the boy who is on the bridge will not get a ball, or, alternatively, he will not get a car, or, possibly he won't get a ball or a car. The results obtained showed that the children with HFA tested in this study were able to use c-command in order to distinguish between sentences where negation only preceded but did not c-command disjunction vs. those where negation both preceded and c-commanded disjunction. In the latter case, the children were able to generate conjunctive entailment consistent with De Morgan's law of propositional logic. If the children with ASD were adopting a linear strategy then they would have attributed disjunctive truth conditions to both sentences.

Notice that the pattern of performance for TD children was more accurate performance on the c-command sentences like (7) than the non c-command ones like (8). This pattern has been observed in other studies too. In Crain et al.'s (2002) study conducted with 4 and 5 year old TD children, the pattern was similar. Children rejected the c-command sentences like (7) 92% of the time while accepted the non c-command sentences less, 87% of the time although there was less difference in the two conditions than in the present experiment. So, why is it that the children are more accurate on the c-command sentences? One possibility that was explored by Gualmini and Crain (2005) was that there is more length between the negation and disjunction operators in the non c-command sentences. They manipulated the number of words between the operators putting more length (5 words) between the operators in the c-command sentences like *Winnie the Pooh will not let Eeyore eat the cookie or the cake* (5 words) and less length (3 words) in the non c-command sentences like *The Karate Man will give the Pooh Bear he cannot lift the honey or the doughnut*. The hypothesis was that if length was the relevant factor, children should perform more poorly on the c-command sentences than the non c-command ones. However, this was not the case. The performance of children was 85% correct on c-command sentences while their performance was 80% correct on the non c-command sentences. The results showed that the interpretation of sentences was not determined by the number of intervening words between negation and disjunction. Consequently, children assigned a conjunctive interpretation only to sentences where negation c-commanded the disjunction.

There is another possible account of the lower accuracy on the non c-command sentences in our experiment with HFA children. This possibility hinges upon differences in the execution of the present study and the studies conducted by Crain et al. (2002) and Gualmini and Crain (2005). Crain et al. (2002) presented the c-command and non c-command sentences in two different sessions, to avoid any carryover effects, and Gualmini and Crain (2005) used a between subjects design. Our experimental design, on the other hand, was a within subjects design, so the participants heard both conditions within the same session. This may have caused some confusion. In addition, some of the non c-command sentences were true and one false, which again, may have meant the responses were less accurate for the non c-command sentences. Nevertheless, the pattern is the same as Crain et al. (2002) experiment, which leads us to infer that children's interpretations are based on computations of hierarchical sentence representations.

Another noteworthy finding from the present investigation is that the children with autism did not appear to have any difficulty processing relative clauses. This result contrasts with the findings of Durrleman and Zufferey (2013) who reported comprehension difficulties for both subject and object relative clauses in HFA French-speaking adults. The present set of sentences only contains subject relatives (e.g., *the boy who is on the bridge will not get a ball or a car*) and it is well known that object gap relative clauses are more challenging, but nevertheless, the children with autism performed well on our task. Our findings are consistent with those reported by Durrleman et al. (2015) who showed that French-speaking adults with ASD are more likely to master subject relative clauses than object relative clauses. The current results are also consistent with the finding that English-speaking teenagers diagnosed with autism made more errors on object relative clauses as opposed to subject relative clauses in a sentence repetition task (Riches et al., 2010).

If children are able to compute hierarchical relations of c-command for sentences containing logical operators such as negation and disjunction, then they should able to use the same relations for interpreting sentences containing reflexives. If variable binding is an issue, though, it is possible that children could perform well on computing c-command with negation and disjunction, but not for Principle A. However, this latter prediction was not borne out for the HFA group of children in our experiment. It could be the case for children with language impairment, but this is yet to be verified. Our experiment found good performance on both experimental tasks, both with negation and disjunction and Principle A. Especially noteworthy is the fact that our current sample of children was younger (age 5;4–12;7) than the groups examined by Perovic et al. (2013a,b) who were between 6 and 18 years of age. Our results are consistent with the performance of Greek children on binding (Terzi et al., 2014). These authors showed that Greek-speaking children diagnosed with autism did not show deficient performance on reflexive binding. It is also reported by Geutjes (2014) that Dutch children diagnosed with autism show performance similar to typically-developing children on Dutch strong and weak reflexives. Although there are language specific differences between Greek, Dutch and English, which may introduce further variables, Principle A is nevertheless a universal principle, and so in principle, there should be no cross linguistic differences (see Thomas, 1991). A thorough cross-linguistic comparison of binding in autism will thus be a fruitful research direction in this regard.

Performance on Control Possessive (CP) sentences was also similar for both the groups of children in our study. Further analysis revealed that the performance on the CP condition was not significantly different from the performance on the Name Reflexive condition (NR) for both the groups. Taken together, our results provide evidence for intact grammar in children on the higher end of the autism spectrum. However, the HFA group performed 15% lower than their typically-developing peers only on the reflexive condition. Thus, we settle for a conservative hypothesis that children with HFA are sensitive to c-command for constraining relations between disjunction and negation. They are also likely to be sensitive to c-command for establishing the complex syntactic dependency of binding instead of relying on a linear strategy. However, in order to pick out the correct antecedent for reflexives, children not only need c-command, they also need to be able to understand that the relationship between the antecedent and the referent is one of variable binding. It may thus be possible that future studies with a larger sample size could show a deficient performance on sentences governed by Principle A for children with autism. This appears to be a plausible interpretation of our results as the performance of the HFA group (85%) was comparable to the performance of the comparison group (83%) on the control sentences (CP). The results hint at the possibility that the complexity of variable binding could pose a problem for children with autism. However, good performance on the CP sentences is not likely to be the result of relying on a linear strategy or choosing the nearest noun as the referent for the antecedent.

### Role played by non-verbal abilities

Our findings are in accord with the latest findings reported by Janke and Perovic (2015). This recent study showed that 26 British HFA children (non-verbal IQ > 80 as assessed by the Matrices subtest of the KBIT) had intact comprehension of reflexives. The authors furthermore classified the children as ALI based on their performance on standardized language tests (the TROG and the British Vocabulary Scales). Only three children, classified as ALI, showed less than perfect performance on reflexives. In other words, the authors noted individual variation. We did not divide our children into a language intact or language impaired category due to the comparatively smaller size of the sample that was only tested on the TROG, although language scores for 6 children from our sample could be considered to be in the impaired range^11^ as they scored below the 10th percentile (e.g., Whitehouse et al., 2008). Thus in the interim, we hypothesize that children with HFA form a distinct linguistic phenotype with respect to intact grammatical functioning (see Perovic et al., 2013b; Janke and Perovic, 2015). It remains to be seen whether children with low-functioning autism (LFA) or those with ALI show any improvements of performance on a different task which has not been usually used, i.e., TVJT^12^. In the meantime, then the general impression seems to be that LFA children will show deficits of syntax. This is because higher non–verbal IQ is an important prognostic variable for clinical populations in general and for autism in particular (Szatmari et al., 1989). Comparatively, LFA children are at a higher risk for language impairment irrespective of the degree of intellectual impairment (Kjelgaard and Tager-Flusberg, 2001).

## Conclusions

In the two experimental studies that we have reported, HFA children did not have difficulty computing the hierarchical relationship of c-command. In sentences containing negation and disjunction, children distinguish the interpretations they allow, depending on whether or not negation c-commands negation. In sentences in which there is a c-command relation between negation and disjunction, children successfully impose a conjunctive entailment, while attributing disjunctive truth conditions to sentences in which c-command does not hold. Furthermore, children are successful in picking out the correct antecedent for the reflexive in sentences like *Bart's dad washed himself with soap*, in conformity with Principle A. It will be instructive to replicate these studies in the future with a larger sample of children, while carefully controlling for HFA children with and without language impairment. This is because studies report different results for HFA children and LFA (Boucher, 2009), or for children classified as ALI vs. ALN (Tager-Flusberg, 2006). Nevertheless, the findings from our English-speaking sample of children concur with the findings of English-speaking British children for binding (Janke and Perovic, 2015). These investigations all suggest that children at the high end of the spectrum may not have any kind of syntactic deficiency (e.g., Terzi et al., 2016a,b). Further cross-linguistic investigation with other complex syntactic structures will be important to shed light on this issue.

## Ethics statement

This study was carried out in accordance with the recommendations of Human Research Ethics Committee at Macquarie University (ref: 5201200880) with written informed consent from all subjects. All subjects gave written informed consent in accordance with the Declaration of Helsinki.The protocol was approved by the Human Research Ethics Committee at Macquarie University.

## Author contributions

RT and NK jointly conceptualized the study and wrote the manuscript. NK collected the data for the study and conducted the analyses.

## Funding

This study was part of NK's PhD thesis titled, “Grammatical Knowledge in Children with Autism.” NK was supported by the International Postgraduate Research Scholarship (IPRS) for the entire tenure of her PhD (2011–2015) at Macquarie University.

### Conflict of interest statement

The authors declare that the research was conducted in the absence of any commercial or financial relationships that could be construed as a potential conflict of interest.

## Footnotes

^1^Grammar here refers to the structural aspects of language, or syntax.

^2^Mixed with this line of argument is the claim that grammar is *relatively* but not *entirely* spared in autism. In other words, the grammatical skills are better than the pragmatic functioning observed for the disorder. For example, Eigsti et al. (2007) observed that children with ASD showed grammatical impairments as compared to groups matched for both non-verbal IQ and receptive vocabulary. Other researchers have found no impairments in grammar when compared to control groups matched for cognitive function, including a Down syndrome group (Tager-Flusberg et al., 1990).

^3^Bishop et al. (2000) and Norbury et al. (2002) recommend a cut-off score of 80 on non-verbal reasoning.

^4^The results from this study indicate that non-verbal abilities cannot straightforwardly account for grammatical difficulties observed in autism as the TD group matched on non-verbal abilities was chronologically younger. However, the findings are consistent with previous studies that matched children with ASD to TD children based on non-verbal abilities (e.g., Perovic et al., 2007). The performance of the TD group developed as a function of their age and non-verbal abilities. On the other hand, the performance of the ASD group developed as a function of only their non-verbal abilities.

^5^The interpretation of reflexives (e.g., himself) and pronouns (e.g., him) is regulated by what is known as Binding Theory (Chomsky, 1981) within the generative approach. The Binding Theory constrains the interpretation of reflexives, pronouns and names through three linguistic principles, known as Principle A, B, and C respectively.

^6^A binds B, if A c-commands B and A and B are coindexed. Reflexives that comply with Principle A are bound pronouns as these are c-commanded by their antecedents and both the reflexive and the antecedent are coindexed and appear in the same clause (e.g., *Sam* in “Sam_i_ washed himself_i_”). Coindexation is shown by indices below the relevant NP.

^7^The delay in understanding pronouns as compared to reflexives has been explained in terms of late developing pragmatic knowledge in children (cf. Chien and Wexler, 1990). Binding principles regulate syntactic binding only. Reflexives are always bound by their antecedent. Binding is also relevant for pronouns, but in addition, the notion of coreference is relevant. One prominent proposal is that children have innate knowledge of Principle B as it regulates whether a pronoun is bound or free in its clause but they have difficulty with coreference as this invokes pragmatic knowledge which is subject to maturation (Chien and Wexler, 1990).

^8^Both reflexives and prnouns can serve as bound variables. To take an example, in the sentence “*Every girl*_i_ *thought she*_*i*/*k*_ *could participate in the competition*,” the pronoun “she” is bound by the quantificational expression “every girl.” In this example, it is easy to see that the pronoun can function as a variable. The sentence is, in fact, ambiguous. If the pronoun “she” is taken to be a referential pronoun, then it refers to some unnamed female individual. In this case, the sentence means that every girl thought that this female individual could participate in the competition. On the alternative interpretation, the pronoun “she” acts as a variable. On this interpretation of the sentence, there are multiple girls, each of whom thinks that she herself could participate in the competition. In a sentence like “Bart's dad is touching himself” the reflexive is a bound variable even though it doesn't pick out a range of individuals. This is simply because the lexical item *himself* is singular. If the sentence was “Bart's kids are touching themselves” then it can be seen that there are multiple individuals who could each be touching themselves.

^9^Whether or not variable binding is problematic for children with autism is an issue for future research. Previous research has shown that typically-developing children as young as 3 and a half years of age can produce bound variable structures. For example, Thornton (1990) elicited questions such as “*Which guys said they have a blue marble*?” in a situation in which each of 3 guys has a marble. In Thornton's study, children often opted for the plural form of the pronoun as the bound pronoun instead of the singular form, *he*.

^10^Recall 3 of the 4 sentences were acceptances, while one was designed to be false and a rejection.

^11^Our sample of HFA children had a low mean SS (76.58) on the TROG while the HFA children examined by Janke and Perovic (2015) had a mean score of 91.73 and those examined by Perovic et al. (2013b) had a mean score of 94.50 on the TROG. However, our children were chronologically younger than those examined by Janke and Perovic (2015) and Perovic et al. (2013b).

^12^Our choice of methodology (a dynamic version of the TVJT) may also have been optimal (see Sanoudaki and Varlokosta, 2015, who demonstrated task effects for the interpretation of Greek strong pronouns).
